# Phase Equilibria, Diffusion and Structure in the Epoxypolycaprolactone System

**DOI:** 10.3390/polym15010117

**Published:** 2022-12-27

**Authors:** Irina O. Plyusnina, Nikita Yu. Budylin, Alexey V. Shapagin

**Affiliations:** Frumkin Institute of Physical Chemistry and Electrochemistry, Russian Academy of Sciences, Leninsky Prospect 31-4, 119071 Moscow, Russia

**Keywords:** DGEBA, polycaprolactone (PCL), phase state diagram, interdiffusion, phase structure, biodegradable material

## Abstract

Currently, there is no quantitative approach for the phase structure of cured thermoplastic systems modified with thermoplastic predicting. To solve this problem, we carried out the first stage of the study on a model polycaprolactone–epoxy oligomer (PCL–DGEBA) system. Using differential scanning calorimetry (DSC), refractometry and optical interferometry, a phase diagram for PCL–DGEBA mixtures was constructed, and the Flory–Huggins interaction parameters of PCL–DGEBA mixtures were calculated. The structure of PCL–DGEBA mixtures with different PCL content was analyzed by optical microscopy. The change in the structure formation mechanism with increasing PCL concentration was shown. The diffusion coefficients are calculated by the Motano–Boltzmann method. The values of the apparent activation energy of the viscous flow PCL and of self-diffusion of DGEBA are determined. The obtained data will be used for the in situ curing kinetics and phase equilibria in the diffusion zone investigations in order to develop a quantitative method for predicting the phase structure of cured systems.

## 1. Introduction

Modification with thermoplastic polymers is widely used to improve their performance characteristics. During the reaction of curing such compositions, the macromolecular mobility of the components decreases, leading to the formation of a heterogeneous structure. In this case, the characteristics of materials are dependent not only on the properties of homopolymers, taking into account their ratio in the mixture, but on the type of phase structure, the nature of the continuous phase, and the size of dispersed particles. It is important that only a qualitative approach for the phase structure prognostication has been considered [1,2,3]; this is based on studying the correlation between the concentrations of the initial components of the composition, curing temperatures, and SEM images of the final phase structures analysis. The technique for determining the quantitative parameters of the initial composition, which make it possible to obtain a given structure of the cured composition, would bring polymer materials science to a new level in the field of modification of reactive systems. In this two-staged work, we will propose a quantitative method for predicting the final phase structure of cured thermoset–thermoplastic compositions. Thermoset materials are used as matrices for polymer fibrous composite materials or in the formation of interpenetrating systems [4] and, due to their densely reticulated topology, are characterized by excellent chemical and biological resistance [5,6].

In this regard, in addition to the fundamental task described above, we will also solve the problem of obtaining composite materials capable of biodegradation and possessing the required performance properties. Such materials include polymer composites containing biodegradable macromolecular components [7]. Note that biodegradation is usually achieved by incorporating labile segments into the polymer matrix that are subject to thermal or hydrolytic cleavage [8,9]. Thus, one of the topical areas of modern polymer materials science is the preparation of composite materials based on large-tonnage polymers that demonstrate a combination of structural properties of components with the possibility of biodegradation.

Bisphenol A diglycidyl ether (DGEBA) diane epoxy resins are widely used thermosets [10]. Among the biodegradable modifiers for DGEBA are vegetable oils [11,12,13], rosin [14,15], lignin, [16] polylactic acid (PLA) [17], and in particular, thermoplastic biopolymers of synthetic origin, such as polycaprolactone (PCL).

PCL is a semi-crystalline polymer that can be degraded by hydrolytic degradation. The inclusion of PCL in a DGEBA matrix is one way to modify an epoxy resin without losing its mechanical properties. Interest in this system is also caused by the fact that such materials, due to the melting and crystallization of PCL, demonstrate the properties of a self-healing material [18,19,20].

It should be noted that most studies of the PCL—epoxy system are aimed at studying the kinetics of the formation of a phase structure during the curing of an epoxy oligomer [21,22,23]. At the same time, there is practically no information on phase equilibria and diffusion upon mixing of the initial components.

Using rigid-chain thermoplastics as an example [24,25,26,27,28,29], it was shown that the remoteness of the critical point of the initial system in the field of the phase diagram from the point of the cured system determines at what degree of conversion of epoxy groups the phase decomposition begins. The degree of conversion affects the macromolecular mobility of the components (diffusion constants) at the beginning of phase decomposition and, consequently, determines the size of the final phase structures. In addition, information on phase equilibria and diffusion constants of initial uncured systems are of fundamental importance for phase structure prediction. Thus, this work represents the first stage of complex research aimed at solving the problem of regulation of the phase structure of cured thermoset polymers modified with thermoplastics. In this regard, the results of studies of compatibility, interdiffusion, and temperature transitions of the components of the PCL–DGEBA compositions in a wide temperature and concentration range are presented in the manuscript. On the basis of the obtained dependences at the second stage, a technique of determining the temperature–concentration parameters, which determine the type of the final phase structure of the cured composition, will be developed. This is of fundamental importance for the performance characteristics of material predicting.

## 2. Materials and Methods

### 2.1. Materials

**Figure 1:**
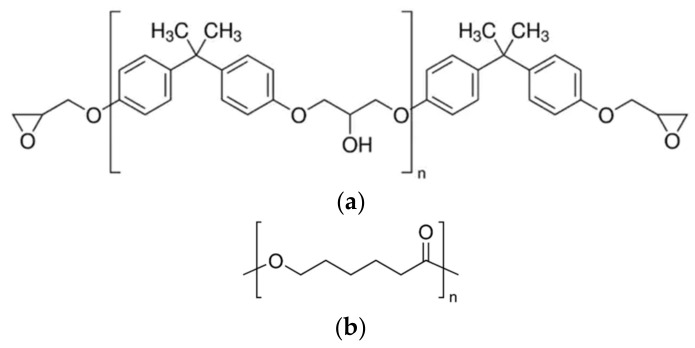
Chemical structures: (**a**) DGEBA, (**b**) PCL.

Epoxy oligomer DGEBA (“Sipo”, Moscow, Russia) is a low molecular weight product of polycondensation of epichlorohydrin with bisphenol A with M_n_ = 380 g/mol, epoxy number 20 ÷ 22. Polycaprolactone (PCL) was by (“Sigma-Aldrich”, Darmstadt, Germany) with molecular weight M_n_ = 45 × 10^3^ and 80 × 10^3^ g/mol. The chemical structures of DGEBA and PCL are shown in Figure 1.

### 2.2. Differential Scanning Calorimetry (DSC)

Thermograms characterizing the melting (T_m_) and crystallization (T_c_) temperatures of PCL, as well as the melting temperatures of PCL–DGEBA mixtures with PCL volume fraction (φ) of 0.38 and 0.67 obtained on a differential scanning calorimeter Netzsch DSC 204F1 Phoenix (“Netzsch-Gerätebau GmbH”, Selb, Germany) [30]. All PCL–DGEBA mixtures were homogenized at 80 °C, transferred to the crucible and then cooled down to 20 °C. The scanning speed in all experiments was 10 K/min.

### 2.3. Optical Interferometry

The study of solubility and interdiffusion in binary systems PCL–DGEBA was carried out by optical interferometry on an ODA-2 IPCE diffusiometer (“IPCE RAS”, Moscow, Russia) [31]. A helium-neon laser (λ = 632.8 nm) was used as a light source.

The method is based on the principle of in situ registration of optical density distribution in the area of conjugation of components and recording its change in time under the isobaric–isothermal conditions of the process [32]. The measurement method consisted in placing a PCL sample of 5 mm × 5 mm in size and about 150 µm thick (obtained by pressing) between the glasses of a diffusion cell with a translucent metal (Ni-Cr alloy) coating with a high reflection index on the inner surfaces. A small wedge angle of 2° was set between the glasses. The assembled cell was thermostated at a given temperature for at least 30 min. Then the space between the panes was filled with DGEBA.

In order to obtain information on phase equilibria, the studies were carried out in the mode of stepwise heating and cooling with a step of 10 °C in the temperature range from 80 to 20 °C. At each stage, the system was thermostated to an equilibrium state, and interferograms were recorded.

Information about the rates of mass transfer processes was obtained in the isothermal holding mode in the temperature range from 80 to 160 °C. Diffusion coefficients were obtained and calculated over the entire concentration range.

**Figure 2:**
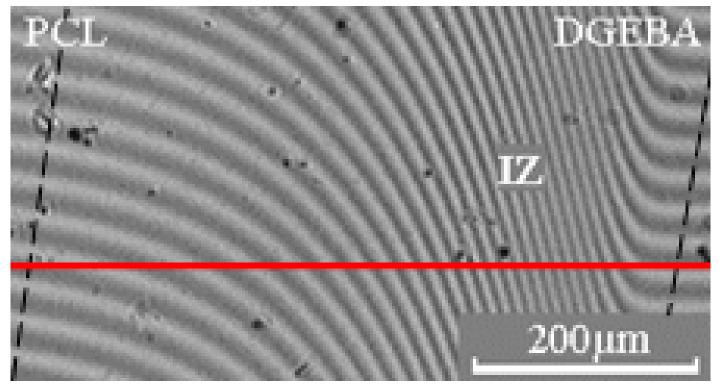
Interferogram of the interdiffusion zone of the PCL–DGEBA system at 80 °C. IZ is the interdiffusion zone.

Methods of processing the interferograms, interdiffusion zones, and phase diagrams construction did not differ from those described earlier [33,34]. In the interdiffusion zone (IZ) (between the dashed lines), a solid line parallel (red line) to the interference fringes in the region of pure components is drawn (Figure 2). The ratio C = 1/N, where N is the number of intersections of the solid line with the interference fringes in the interdiffusion zone, equals the concentration increment when passing from one interference fringe to another. The compositions of coexisting phases were calculated using the formula C_i_ = C × N_i_, where N_i_ is the number of crossings of the solid line with interference fringes to the left (or right) of the phase boundary or heterogeneous zone.

### 2.4. Refractometry

To construct a phase diagram from interferograms, the temperature dependencies of the refractive index of the components are required. The difference in refractive indices between the components of a diffusion system determines the total number of interference lines (N), the increment of the refractive index, and the concentration per line (Δ):

(1) $$N=\frac{n_{1}-n_{2}}{\Delta }$$

For this purpose, studies were carried out on an Abbe refractometer ATAGO NAR-2T (“Atago Co. Ltd”., Tokyo, Japan) in the stepwise cooling and heating mode in the range from 80 to 20 °C with an accuracy of ±0.0001, covering the temperature range of diffusion measurements and phase transitions (in this case, T_m_ and T_c_).

The PCL film was placed between two prisms, heated above the melting point of PCL to 100 °C, then cooled down to 20 °C stepwise with a thermostating time at each temperature of at least 20 min. The studies were carried out in the “heating–cooling” cycle, which made it possible to identify non-equilibria that may arise during the crystallization and melting of the polymer.

### 2.5. Optical Microscopy

To study the phase structure of the PCL –DGEBA system, polarizing optical microscopy Olympus BX 51 (“Olympus”, Tokyo, Japan) was used.

The experimental procedure was as follows. The test mixture of a given composition was homogenized at a temperature above the melting point of PCL 100 °C, after which the mixture was evacuated for 2 h. Then, a sample of the melt was applied onto a heated glass slide and placed on the microscope stage. Cooling was carried out at a rate of 10 °C/min. Structure images were fixed at room temperature.

## 3. Results and Discussion

**Figure 3:**
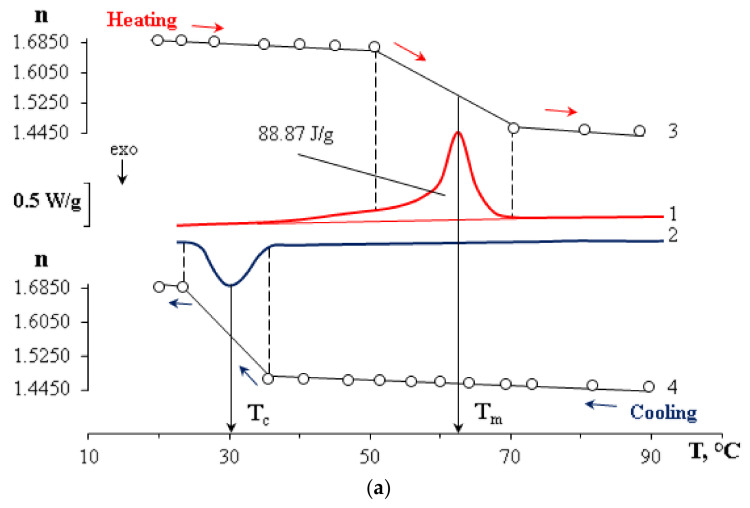

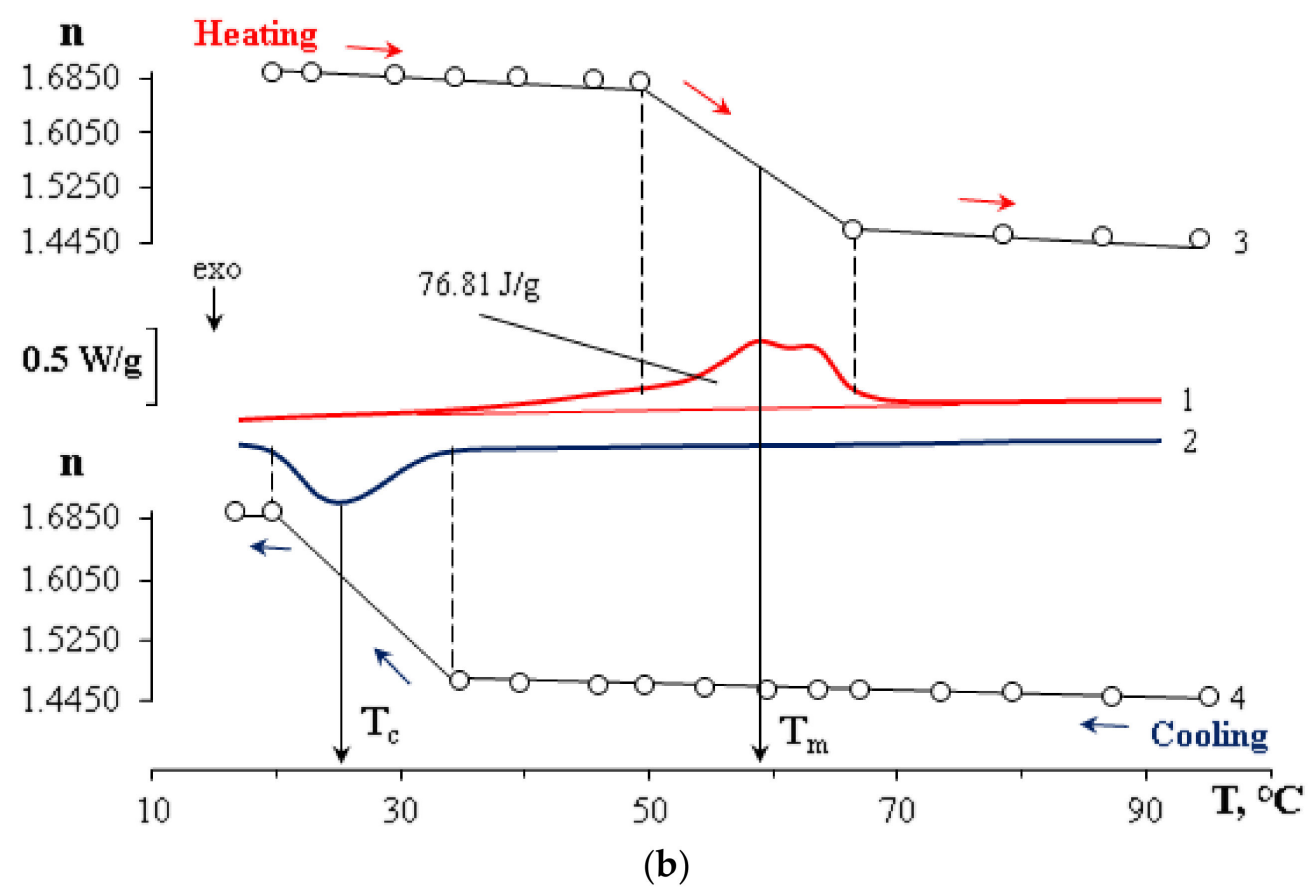
DSC thermograms in heating mode (1), cooling mode (2), and refractometric curves in heating mode (3) and cooling mode (4) for PCL with (**a**) M_n_ = 45 × 10^3^ and PCL with (**b**) M_n_ = 80 × 10^3^.

DSC and refractometry were used to obtain phase transition temperatures (melting and crystallization) for polycaprolactones of two molecular weights. For M_n_ 45 × 10^3^ g/mol T_m_ = 62.9 °C, T_c_ = 30.1 °C, for M_n_ 80 × 10^3^ g/mol the melting temperature range is identified by three fractions with the largest contribution at T_m_ = 59.3 °C, T_c_ = 25.1 °C (Figure 3). PCL with lower M_n_ is characterized by more regular crystal structures, which leads to an increase in their size and an increase in the melting temperature (Figure 3a). As the molecular weight increases, the enthalpy peak broadens on the DSC thermogram (Figure 3b), the defectiveness of crystal structures increases, and the melting temperature decreases.

**Figure 4:**
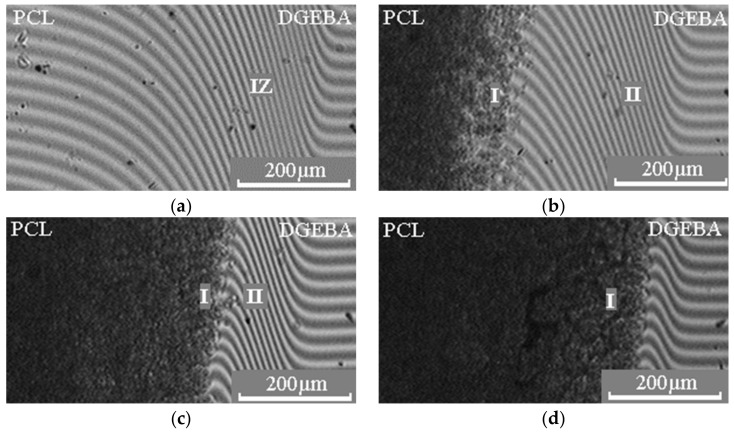
Interferograms of the interdiffusion zone of the PCL (M_n_ = 45 × 10^3^) – DGEBA system, obtained at temperatures: (**a**) 80; (**b**) 40; (**c**) 30; (**d**) 20 °C. IZ is the interdiffusion zone, I is the zone of DGEBA diffusion in PCL, II is the zone of PCL diffusion in DGEBA.

Diffusion zones in binary gradient solutions–melts of the PCL –DGEBA system were studied (Figure 4). It is shown that above the melting point of PCL, in the interdiffusion zone of this system, we observed the formation of a concentration profile with a continuous change of composition from one component to another (Figure 4a). With a decrease in temperature in the systems, crystallization of PCL dissolved in the epoxy oligomer occurs (Figure 4b–d). In this case, the compositions of the coexisting phases are a saturated solution of PCL in DGEBA and crystals (spherulites) of PCL. A similar nature of the interferograms is also observed in the PCL (M_n_ = 80 × 10^3^) – DGEBA system.

**Figure 5:**
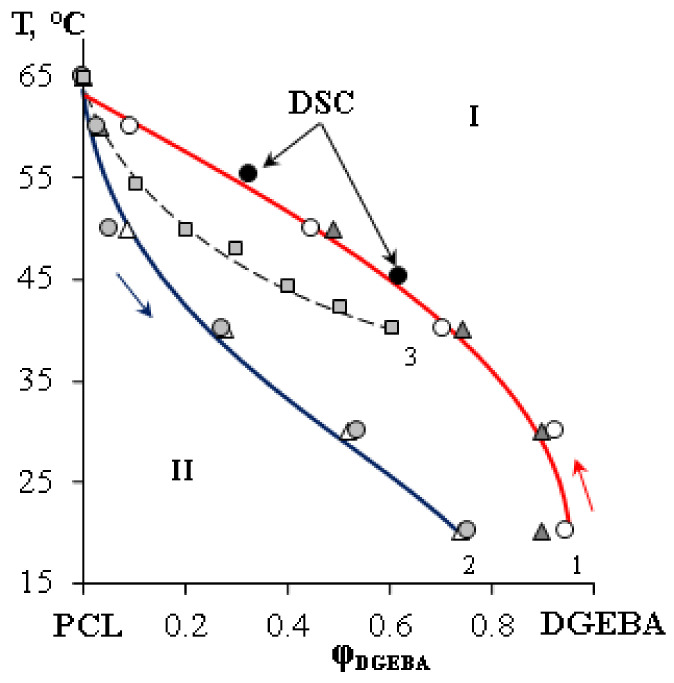
Phase state diagram of the PCL–DGEBA system, where 1—liquidus line, 2—solidus line, 3—literature data [35]. 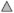, 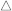 —compositions of coexisting phases for M_n_ PCL = 80 × 10^3^; 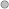, 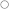
—for M_n_ PCL = 45 × 10^3^. 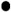
—melting points of the PCL–DGEBA mixture (M_n_ PCL = 45 × 10^3^) obtained by DSC. I—homogeneous solution; II—heterogeneous solution.

An analysis of the diffusion zones in the heating and cooling regimes made it possible to calculate the liquidus and solidus lines of the studying systems and construct a phase state diagram of the crystalline type (Figure 5). The values of the coexisting phase compositions are in equilibrium, since they are reproduced in a cyclic mode.

It can be seen from the phase diagram that with an increase of the DGEBA content in the mixture, the melting point is depressed. It is weakly expressed in the concentrations range close to PCL and is significant in the dilute solutions range. Note that the difference between the melting and crystallization temperatures reaches 20 °C. It has been established that an increase in the molecular weight of PCL does not make a significant contribution to the composition of coexisting phases.

**Figure 6:**
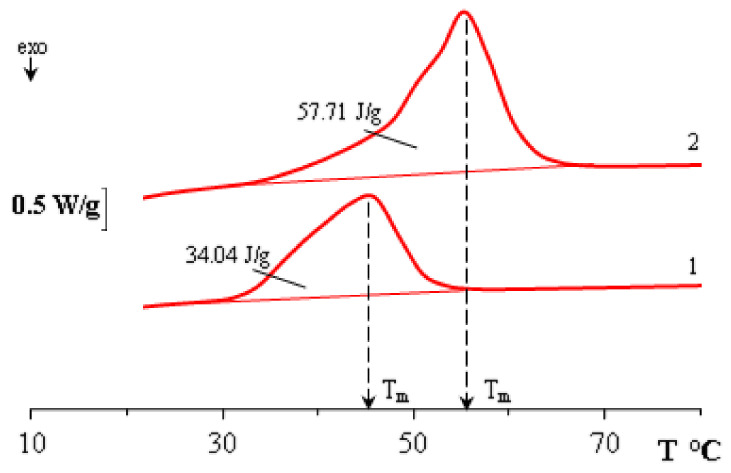
DSC thermogram of mixtures containing PCL (M_n_ = 45 × 10^3^): 1 – φ_PCL_ = 0.38, 2 – φ_PCL_ = 0.67.

To confirm the position of the liquidus line in the temperature–concentration field of the phase state diagram corresponding to the PCL with M_n_ = 45 × 10^3^, mixtures were prepared with a PCL content φ = 0.38 and 0.67 vol. d. and studied by DSC (Figure 6). The obtained values of the melting points of the mixtures clearly fall on the liquidus line determined by optical interferometry (Figure 5). It can be seen that an increase in the content of PCL in the mixture contributes to the broadening of the melting peak and, as a consequence, to an increase in the polycrystallinity of the formed structures. Note that the obtained points on the phase diagram correlate with the literature data [35].

From the depression of the melting temperatures of PCL, using Equation (2), the pair parameters of the interaction of DGEBA with PCL were calculated (Table 1):

(2) $$\chi =\frac{\frac{-{\Delta H}_{m}^{o}}{{\mathrm{RT}}_{\mathrm{mix}}}\left(1-\frac{T_{\mathrm{mix}}}{T{}^{\circ}}\right)-\frac{\ln\left(\varphi_{2}\right)}{r_{2}}-\left(\frac{1}{r_{2}}-\frac{1}{r_{1}}\right)\varphi_{1}}{\varphi_{1}}$$

where ${\Delta H}_{m}^{o}$ is the heat of fusion of 100% PCL 139.3 J/g [36,37,38], T° is the equilibrium melting temperature of a pure crystallizing component, $T_{\mathrm{mix}}$ is the equilibrium melting temperature of a mixture of components, $r_{1}$ and $r_{2}$ are the degrees of polymerization of DGEBA and PCL [39].

The interaction of the mixture components is determined to a decisive extent by the temperature dependence χ [40]. It is also known that the Huggins parameter for polymer–solvent systems changes with a variation in the concentration of polymers [41].

The obtained values of the pair parameter of the interaction of the analyzed system are positive in all cases and depend on the concentration. A decrease in the content of PCL in the mixture leads to a decrease in χ ≤ 0.5 (corresponds to Θ-conditions). This means that the interaction of PCL segments with DGEBA is energetically favorable at lower PCL concentrations in the mixture.

**Figure 7:**
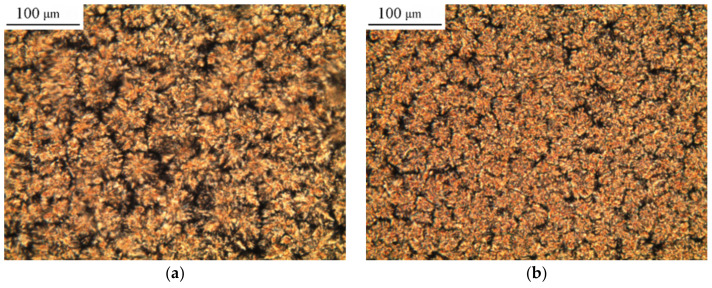

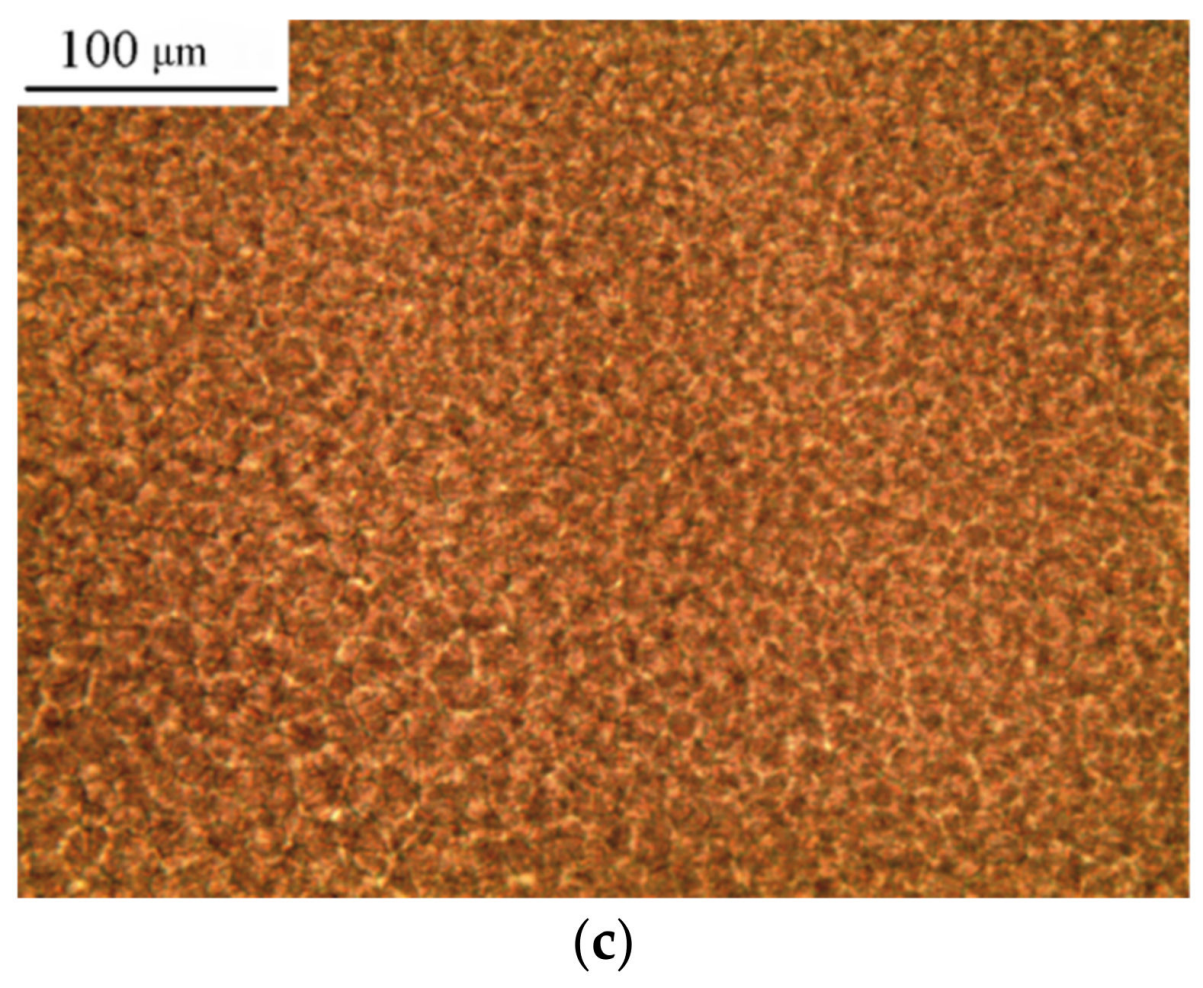
Polarized optical micrographs of PCL (M_n_ = 45 × 10^3^) – DGEBA mixtures with φ_PCL_ (**a**) 0.23; (**b**) 0.38; (**c**) 0.52.

In situ studies by optical microscopy in polarized light showed that mixtures with a PCL content (M_n_ = 45 × 10^3^) of less than φ = 0.13 vol.d. remain transparent at a temperature of 20 °C, while in mixtures with a PCL content of more than φ = 0.13 vol.d. it crystallized (Figure 7). When the samples are heated, the crystal structure disappears, and near the melting point of PCL, the material becomes completely amorphous. The content of PCL in the mixture is less than φ = 0.13 vol.d., which leads to the fact that a homogeneous dilute solution is characterized by a non-uniform distribution of segments over the volume of the system [42]. Individual molecules are isolated from each other; therefore, there are regions of the solution in which the polymer is absent.

**Figure 8:**
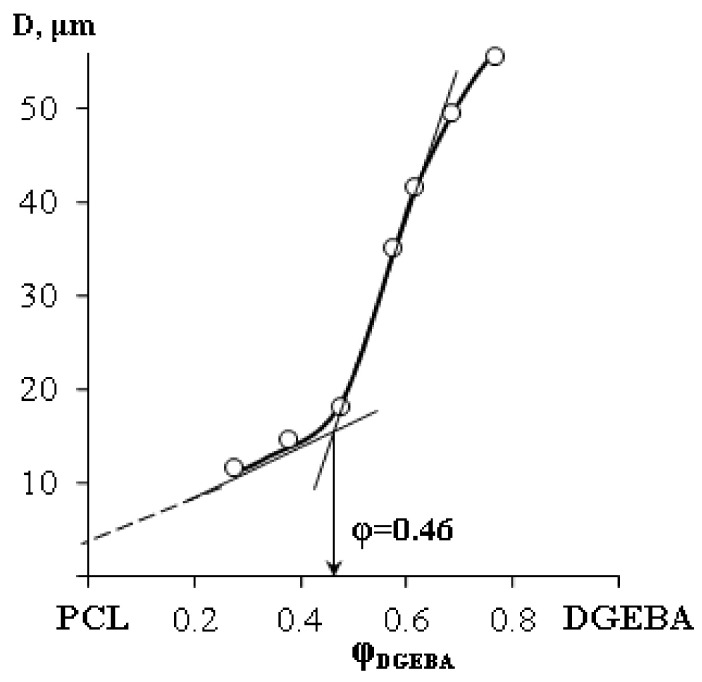
The concentration dependence of the size of crystalline formations in PCL–DGEBA mixtures.

The numerical values of the sizes of crystalline formations (spherulites) obtained after processing microphotographs are shown in Figure 8. It was found that with an increase in the PCL content at φ~0.5 vol.d., the mechanism of structure formation changes and the diameter of spherulites decreases. This is due to an increase in nucleation centers and, as a consequence, a decrease in the distances between them. Another reason for the formation of smaller spherulites at an increased PCL concentration in the mixture is the increase in the difference between the crystallization temperature and the room temperature of the experiment, which is accompanied by an increase in the structure fixation rate.

**Figure 9:**
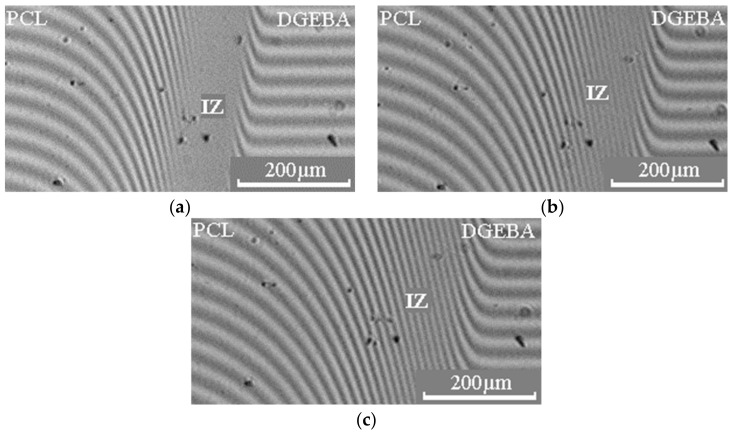
Interferograms of the interdiffusion zone of the PCL (M_n_ = 45 × 10^3^) – DGEBA system, obtained at a temperature of 80 °C for (**a**) 16; (**b**) 25; (**c**) 36 min.

When studying the formation of a phase structure in multicomponent polymer systems, not only information about phase equilibria is of great importance, but also information about the interdiffusion of components in a mixture. Figure 9 shows the interferograms of the interdiffusion zones of the PCL–DGEBA system. The moment of contact between the fronts of the components was considered the beginning of the interdiffusion process.

**Figure 10:**
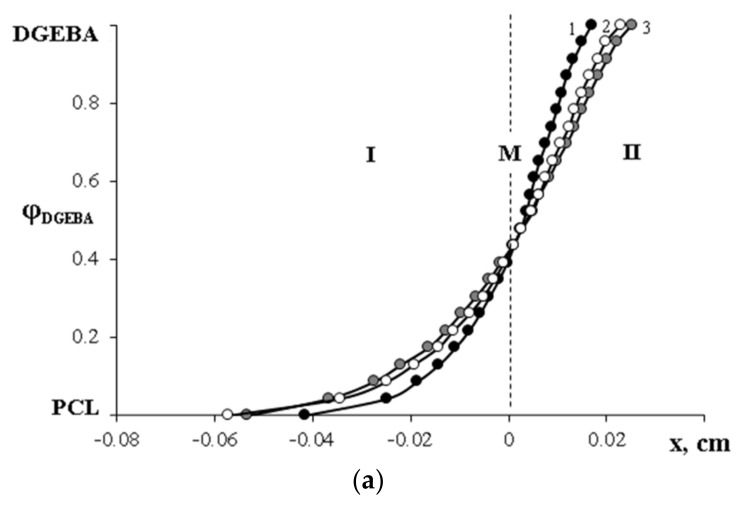

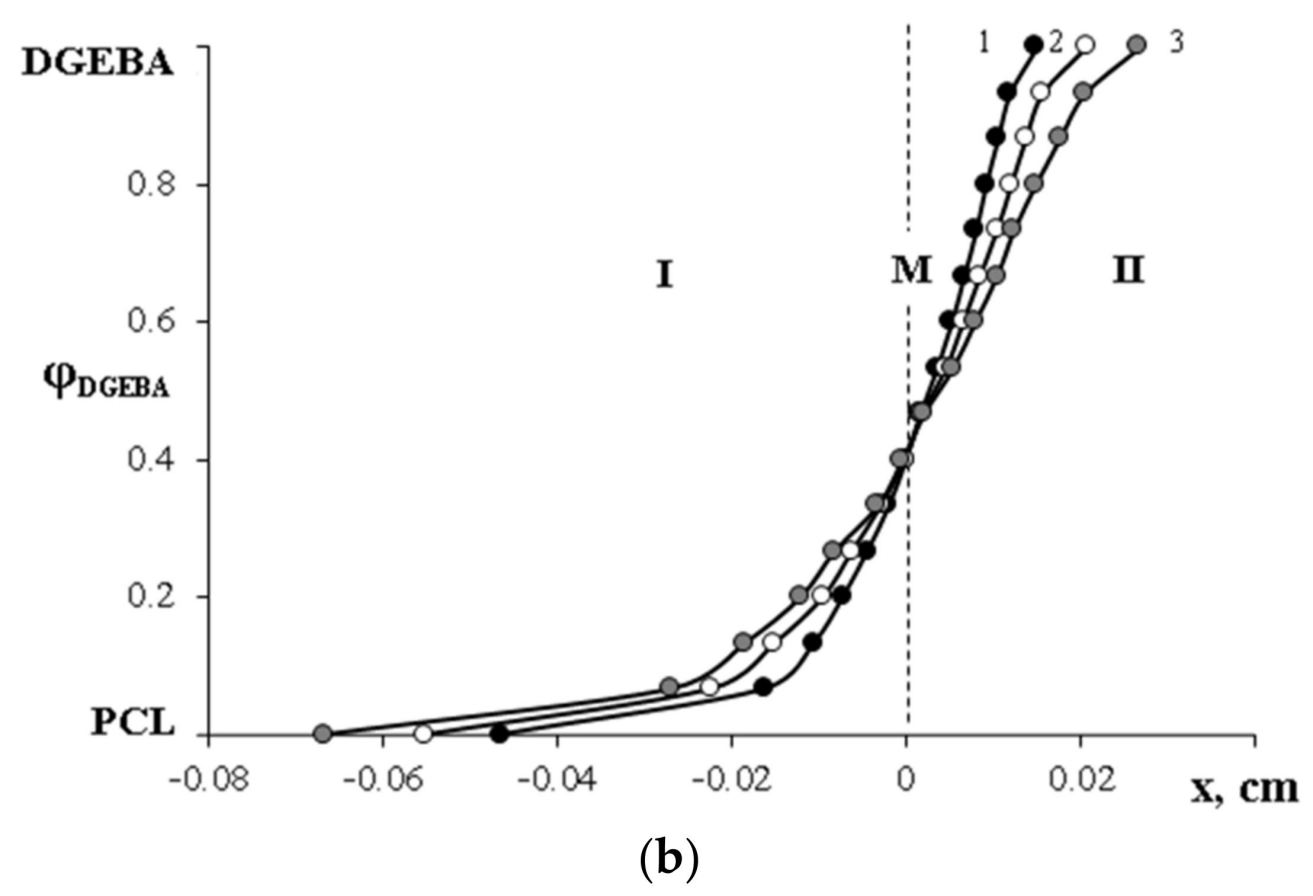
The concentration profiles of PCL–DGEBA systems at 80 °C: (**a**) PCL with M_n_ = 45 × 10^3^, where 1—16, 2—25 and 3—36 min; (**b**) PCL with M_n_ = 80 × 10^3^, where 1—9, 2—16 and 3—25 min. M is the Matano–Boltzmann plane. I corresponds to the diffusion front of DGEBA in PCL, II corresponds to the diffusion front of PCL in DGEBA.

All interferograms at phase conjugation above T_m_ PCL testify to the complete solubility of the components in each other. At the same time, in the interdiffusion zone of the system, the formation of a concentration profile with a continuous change in composition from one component to another is observed (Figure 10). It can be seen that the position of the Matano–Boltzmann plane is constant and corresponds to the average region of compositions. This experimental fact allows us to state that the mixing of PCL with DGEBA occurs without a change in volume.

**Figure 11:**
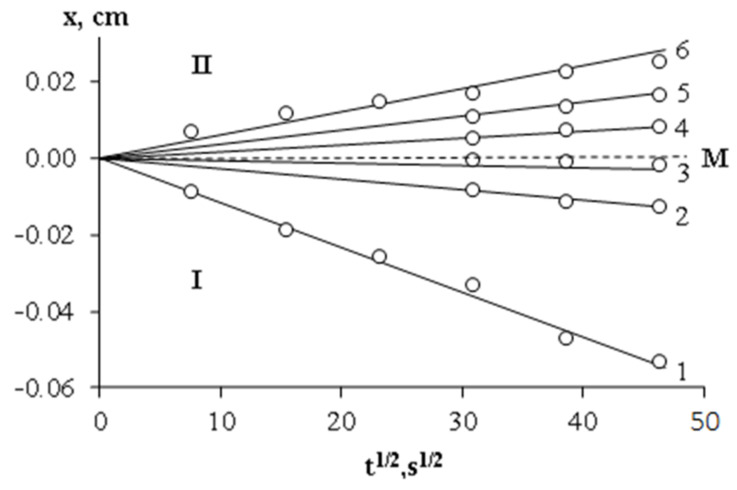
The kinetic dependences of the movements of isoconcentration planes in the PCL (M_n_ = 45 × 10^3^) – DGEBA system at 80 °C. PCL concentrations are indicated near the straight lines. M is the Motano–Boltzmann plane, I corresponds to the diffusion zone of PCL in DGEBA, II corresponds to the diffusion zone of DGEBA in PCL.

Fundamental importance for the analysis of the mixing mechanism of components is information on the motion kinetics of isoconcentration planes in the interdiffusion zone. Figure 11, using the example of a system with PCL M_n_ = 45 × 10^3^, shows typical kinetic dependences characterizing the movement of PCL macromolecules into the DGEBA matrix and the movement of DGEBA molecules into the PCL matrix. The motion of isoconcentration planes in the interdiffusion zone in the coordinates X ≈ k√t, where k is a constant characterizing the partial diffusion coefficients of the components, is described by a linear dependence. A similar character of the movement of isoconcentration planes was observed in the entire selected temperature range. This allows us to state that in PCL-DGEBA systems the process of spontaneous mixing obeys diffusion laws.

**Figure 12:**
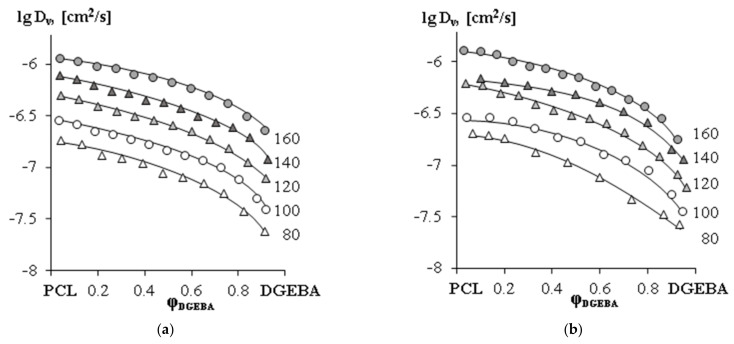
The concentration dependences of interdiffusion coefficients for (**a**) PCL (M_n_ = 45 × 10^3^) –DGEBA system; (**b**) PCL (M_n_ = 80 × 10^3^) — DGEBA system. The temperatures (°C) are indicated near the curves.

It was found that for the studied systems with different M_n_ PCL, there is a common trend in the change in diffusion coefficients with concentration (Figure 12). The value of D_v_ changes smoothly and monotonously when passing from one component to another. The system is characterized by a rather high diffusion mobility of the components. Self-diffusion coefficients of PCL from 10^−6^ to 10^−7^ cm^2^ s^−1^, DGEBA from 10^−7^ to 10^−8^ cm^2^ s^−1^ in the temperature range 80–160 °C. Note that an increase in the PCL molecular weight leads to a slight decrease in the interdiffusion coefficients.

**Figure 13:**
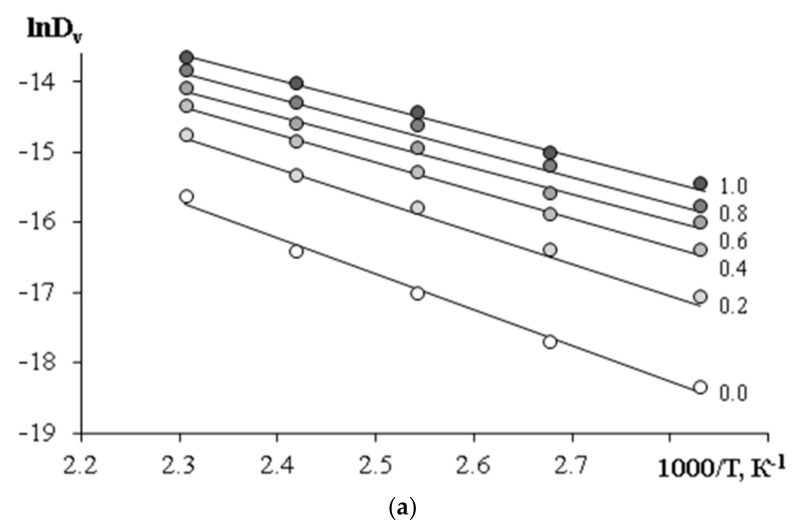

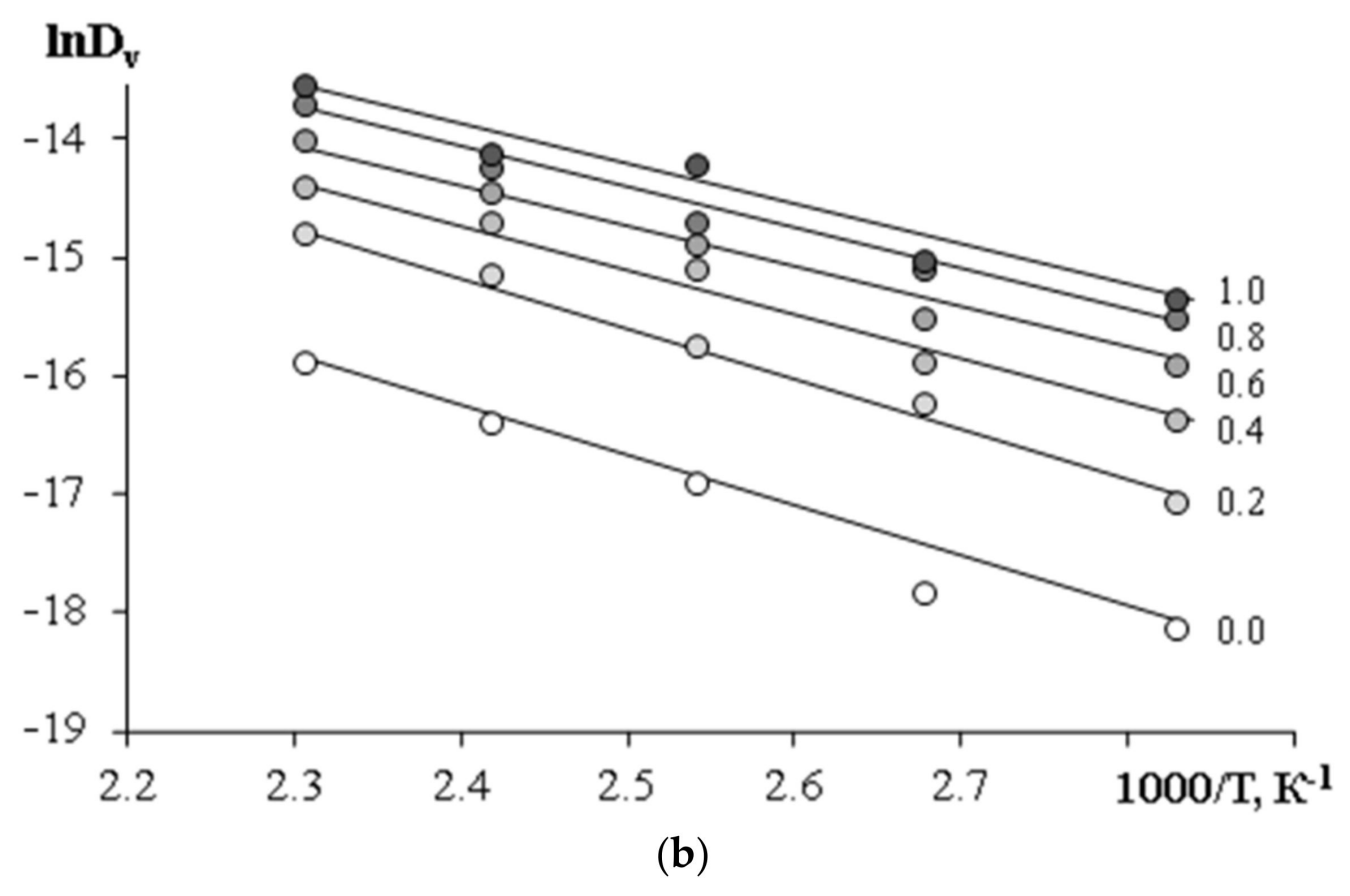
The temperature dependences of lnD_v_ for PCL–DGEBA mixtures: (**a**) M_n_ = 45 × 10^3^; (**b**) M_n_ = 80 × 10^3^. PCL concentrations are indicated near the straight lines.

**Figure 14:**
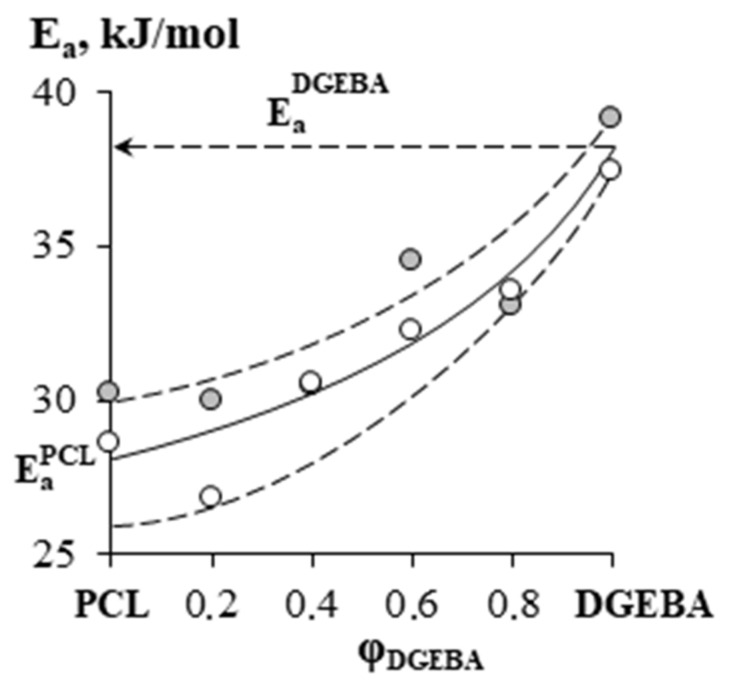
The concentration dependence of the apparent activation energy of interdiffusion in the PCL–DGEBA system, where: 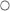
—PCL with M_n_ = 45 × 10^3^, 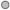
—PCL with M_n_ = 80 × 10^3^.

To calculate the apparent activation energy of the interdiffusion of the components in the coordinates of the Arrhenius equation, the temperature dependences of the diffusion coefficients were plotted (Figure 13). It can be seen that these dependences are linear. The concentration dependences of the apparent activation energy of interdiffusion systems are shown in Figure 14.

It has been established that the apparent diffusion activation energy upon passing from one component to another gradually decreases in PCL-concentrated solutions from the activation energy of self-diffusion DGEBA (38 kJ/mol) to the activation energy of viscous flow of melts PCL 27 kJ/mol for PCL with M_n_ = 45 × 10^3^ and 30 kJ/mol for PCL with M_n_ = 80 × 10^3^ [43].

## 4. Conclusions

DSC studies of pure PCL showed that increasing the molecular weight was accompanied by decreasing melting temperature (~4 °C) and melting enthalpy peak widening. This can be explained by an increase in the defectiveness of crystal structures.

The liquidus and solidus lines of crystal equilibrium phase state diagrams were determined. It was found that in the region of dilute DGEBA solutions in PCL–DGEBA mixtures melting temperature depression occurs. Diluted PCL solutions (up to 0.2 vol.d.) are homogeneous below 40 °C. It can be explained by the heterogeneous volume distribution of the segments. Increasing the PCL content leads to the crystallization of the composition. The size of spherulites and the mechanism of structure formation (at ~0.5 vol.d of PCL) depend on the content of PCL in the mixture. It is shown that PCL and DGEBA are fully observed above the melting point of PCL (66 °C). The diffusion coefficient values lie between 10^−6^ and 10^−8^ cm^2^ s^−1^, which indicates high mobility of the components. It has been found that the apparent activation energy of diffusion for mixtures smoothly decreases from Ea DGEBA (38 kJ/mol) to Ea PCl (27 kJ/mol).

The data obtained will be used in the second stage of our work and will be useful to develop a quantitative technique for predicting the phase structure of hardened systems.

## Figures and Tables

**Table 1:** Values of the Flory–Huggins pair interaction parameter calculated from the melting point depression of PCL in the presence of DGEBA.

T^−1^, K	Χ	
	PCL (M_n_ = 45 × 10^3^) – DGEBA	PCL (M_n_ = 80 × 10^3^) – DGEBA
3.095975	0.538435	0.836821
3.194888	0.228042	0.253214
3.300330	0.093582	0.067616

## Data Availability

Not applicable.
